# A lateral tension model for mouse cranial neural tube closure

**DOI:** 10.1101/2025.05.15.654327

**Published:** 2025-05-15

**Authors:** Juana De La O, Gabriel L. Galea, Adam C. Martin

**Affiliations:** 1 Department of Biology, Massachusetts Institute of Technology, Cambridge, MA 02142, USA,; 2 Department of Developmental Biology and Cancer Researching and Teaching, University College London Great Ormond Street Institute of Child Health, WC1N 1 EH London, United Kingdom,

## Abstract

Organisms mold their tissues into increasingly more complicated shapes during development using a set of deformation motifs. One set of motifs are tissue hinges and creases which are created through cell-level shape changes. Hinges and creases are often made from active, anisotropic cell constriction at the hinge, which can help drive tissue folding. This contractile hinge model is observed in a variety of developmental tissue folding contexts, like during neural tube closure (NTC) of commonly studied species. However, patterns of cells constriction are inconsistent with this model, in the mouse brain. Additionally, formation of the midline neural tube hinge and crease are insensitive to the molecular machinery needed to induce cell shape changes. Here, we test if a contractile hinge is the driving mechanism for mouse cranial NTC. Through targeted laser ablations, we infer tissue tension in the folding mouse cranial neural tube. In contrast to predications of the contractile hinge model, we find the midline hinge has relatively low and isotropic tension, the lateral neural folds have higher anisotropic tension. We also show that regional patterns of tension vary by sex. We propose a lateral tension model for mouse cranial NTC and theorize on the connection between tissue mechanics and sex in NTC defects.

## INTRODUCTION

As organisms develop, they continually sculpt their tissues into more complex three-dimensional shapes. This process of creating shapes, morphogenesis, requires the precise spatial application of forces. Yet, while we observe a menagerie of shapes across developmental systems, a common set of folding mechanisms are repurposed to create them. One example is the formation of hinges and creases. In epithelial tissues, a hinge is a bend that other regions of the tissue move relative to. When a hinge is propagated along the length of a tissue in a line, a crease is formed. These tissue-level structures come about from cell-level shape changes^1^. At the hinge, cells change from a columnar shape to a wedge creating a bend in the tissue. Cell wedging can be enabled by the activity of apically localized filamentous actin combined with non-muscle myosin II, collectively termed actomyosin. The actomyosin anchors to membrane proteins and selectively constricts the apical surface^2^. To form a crease along the length of the tissue, cells at the hinge typically undergo anisotropic wedging, with the greatest apical constriction orthogonal to the length of the crease^3,4^. If cells constrict isotropically, they create a circular pit, like those seen in the optic cups and intestinal crypts^5,6^. In this contractile hinge model, contractility at the hinge and crease is a primary driver for folding of the whole tissue.

Neural tube closure (NTC), an iconic example of tissue folding, displays many of the conserved features of the contractile hinge mechanism. During NTC, a flat epithelium, the neural plate, forms a midline hinge and crease. The lateral neural folds then elevate relative to the midline, and finally opposing edges meet and fuse to form the neural tube (NT), the precursor to the brain and spinal cord^7^. The cranial NT region requires coordinated actomyosin contractility, as genetic or chemical perturbations of actomyosin or its regulators prevents NTC^8^. In several species, a contractile hinge is used to promote NTC. *Xenopus* uses interspersed cells that constrict isotropically or elongate directionally at the midline to fold the NT^9–11^. In the chicken, polarized actomyosin contractility promotes anisotropic constriction at the midline hinge^4^.

At first glance, one might expect the mammalian NT to also employ the contractile hinge mechanism; it also possesses a midline hinge and NTC is sensitive to actomyosin perturbations. However, looking beyond superficial similarities, we find some key discrepancies. Unlike other organisms, the mouse NT adopts geometries that are seemingly antagonistic to closure, especially in the cranial region^12^. The lateral neural folds bulge dorsally, which would interfere with apposition and fusion of the edges. The mouse NT also has global curvature from flexures along the rostral-caudal axis. Additionally, while actomyosin perturbations do prevent full NTC, they do not prevent the formation of the midline hinge or crease^13–16^. In fact, it is the lateral cells, not midline cells, that undergo substantial apical constriction over the course of cranial NTC^17^. A contractile hinge seems to be insufficient to resolve these mechanical constraints and is incapable of explaining behaviors at the midline hinge. What then is the mechanical mechanism employed used to bend the cranial mouse NT?

Here, we set out to interrogate the mechanics of the mouse cranial NT. Through a series of targeted laser ablation experiments in live mouse embryos, we measured initial recoil velocity, a proxy for tension, in discrete regions and orientations of the folding NT. We find that, in contrast to expectations of the contractile hinge model, tension in the mouse NT is higher in the lateral neural folds and lower at the midline hinge. Additionally, the midline hinge has isotropic tension while lateral tension is anisotropic, with the greatest tension along the medial-lateral axis. Finally, we find that regional tension patterns differ between female and male embryos. We propose that the cranial mouse NT uses a lateral tension based mechanical model for closure and speculate on the implications of sex difference during this process.

## RESULTS

**Figure 1:**
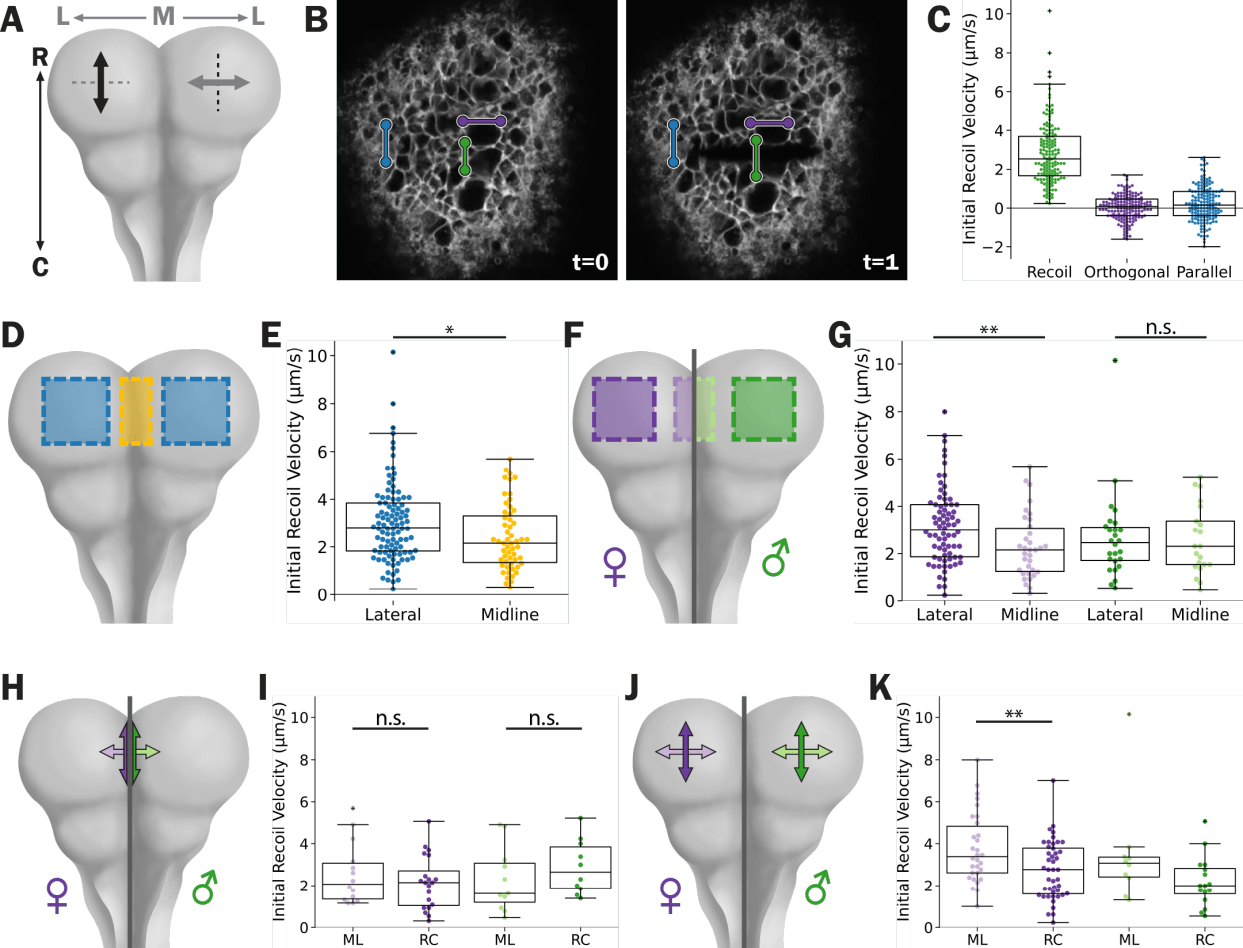
The lateral neural folds exhibit regionally anisotropic tension in a sex-dependent matter. **A, D, F, H, and J)** A cartoon schematic of laser ablation on live mouse embryos. **A)** Cuts (dashed lines) made along one axis allow us to infer tension (double headed arrow) in the orthogonal axis. **B)** Representative images of a CellMask stained embryo before (t=0) and immediately after ablation (t=1). Two points on opposing sides (green) of the ablation were tracked and used to calculate the initial recoil velocity (**C**). Points orthogonal (purple), and parallel (blue) to the ablation serve as internal controls. **D** and **F)** Shaded areas indicate regions where initial recoil measurements plotted in **E)** and **G)** where measured. **H** and **J)** Double headed arrows indicate the direction inferred tension was measured in the **I)** midline and **J)** lateral neural folds. In **F-K)** female measurements are in purple hues and males are in green hues. In **C)**, **E)**, **G)**, **I)** and **K)** box plots show the median and interquartile range. Dorsal (D), ventral (V), rostral (R), caudal (C), lateral (L), medial (M), medial-lateral (ML), rostral-caudal (RC). Not significant (n.s), * p < 0.05, ** p < 0.01.

We used two-photon laser-ablation to interrogate tension in the mouse cranial NT^18^. We measured a proxy for tissue tension by measuring the velocity of tissue recoil after ablation as a proxy for tissue tension (Figure 1A); the faster the recoil orthogonal to the ablation, the greater tension was present prior to ablation. Recoil velocity is influenced by the tissue viscosity and elasticity, dampening over time^3,19^. Therefore, we measured the initial recoil velocity (IRV), defined as the recoil velocity from the time of the ablation to the next immediate frame. We tracked points across the center of the ablation path to calculate the IRV (Figure 1B). We also tracked points orthogonal and parallel to the direction of recoil to control for artifactual background movement, primarily from the embryonic heartbeat. Our recoil measurements averaged ~2.8 μm/s, while both artifactual controls averaged close to 0 μm/s (Figure 1C). We concluded that our assay has sufficient sensitivity to measure meaningful tissue recoils.

We first asked if we could detect regional differences in IRV between the lateral neural folds and midline hinge. The contractile hinge model predicts that tension should be relatively higher at the hinge than the adjacent tissues. It came as a surprise to us to find that, on average, the lateral neural folds have a higher IRV (3.03 μm/s) than the midline hinge (2.40 μm/s, Figure 1D and E). Strikingly, when we disaggregated the data by sex, we found that females had a significantly higher IRV laterally than at the midline (3.15 μm/s vs 2.30 μm/s, 37% difference), while no significant difference was detected between these regions in male (2.68 μm/s vs 2.57 μm/s, 4% difference, Figure 1F and G). For all our measurements, we found no difference in IRV over the developmental stages analyzed (Supplemental Figure 1). These results are inconsistent with the contractile hinge model in the mouse cranial NT.

We next asked if there were directional differences in IRV within regions of the NT. The contractile hinge model requires anisotropic constriction to form a crease. This would result in anisotropic tension in the crease, generally along the length of the crease^20,21^. As we performed ablations, we ensured that all ablations were oriented along either the rostral-caudal (RC) or medial-lateral (ML) axis of the embryo. The resulting IRVs provide information on the tension in the ML and RC axis, respectively (Figure 1A). For clarity, we will exclusively discuss the direction of measured IRVs, and therefore inferred tension, for the remainder of the manuscript. We were again surprised when neither sex had significant IRV anisotropy at the midline hinge (Figure 1H and I). When we examined the lateral neural folds, we found that females have higher ML than RC IRV (3.77 μm/s vs. 2.73 μm/s, 38% difference, Figure 1J and K). We also observed that males appear to have higher ML than RC IRV (3.45 μm/s vs. 2.19 μm/s, 56% difference, Figure 1J and K), but fail to reach significance, potentially due to the smaller sample size. Collectively, our findings suggest that the cranial mouse midline hinge lacks the anisotropic tension that drives tissue folding in the contractile hinge model. If not through the contractile hinge model, how then are these regional tension patterns used to promote NTC?

## DISCUSSION

### A lateral tension model for cranial mouse neural tube closure

In our search for the mechanical mechanisms governing cranial mouse NTC, we have made several key observations that are inconsistent with the contractile hinge model. First, we find that initial recoil velocities, and by proxy tissue tension, are highest not at the midline hinge but at the lateral neural folds. Second, despite the formation of a crease, indicating anisotropic forces at play in folding the neural fold, we find that tension is isotropic at the midline. To this we add two key results from prior literature. First, cells at the middle hinge are larger than lateral cells and do not undergo substantial apical constriction during NTC^17^. Second, perturbations of actomyosin, which promotes apical constriction, does not preclude hinge and crease formation^13–16^. Therefore, we conclude that a contractile hinge is unlikely to drive folding of the mouse cranial NT.

Instead, we propose a lateral tension model for cranial mouse NTC. In this model, high lateral constriction introduces high, directional tissue tension. Combined with previously reported tissue thickening^12^, the lateral tissue becomes rigid and stiff^22^. In contrast, the midline cells, which do not substantially constrict and slightly flatten over time, remain more flexible and amenable to bending. In essence, the neural tube has many of the same characteristics as a hard cover book. This regionalized patterning of tissue tension and rigidity primes the neural tissue to fold correctly in response to external pushing forces. The rigid lateral folds (the book covers) can efficiently elevate, while the flexible midline (the book spine) bends, creating a hinge and crease.

Not only does our model provide a conceptual basis for understanding how NTC proceeds, but it also provides some explanation for some of the previously noted discrepancies. The lateral neural folds bulge dorsally during the initial stages of NTC^12^. Left as they are, these bulges would bump into each other as the neural folds elevate and prevent the apposition and fusion of the edges. Increased apical tension on the lateral folds likely contributes to flattening and ultimate reversal of curvature to enable full closure. Regarding hinge formation in the absence of proper actomyosin activity, our model also provides some insight into this behavior. Rather than forming from active apical constriction or elevated contractility, our model suggests that the midline hinge and crease form as a result from forces extrinsic to the neural tissue that are independent of actomyosin activity. However, it remains possible that other tissue intrinsic cellular activities in the midline hinge contribute to tissue folding.

Our model presupposes the existence of an external force “pushing” on the neural tissue to promote elevation and midline bending. Where is this force coming from? We propose two likely, but not mutually exclusive, sources. First, is the mesenchyme, composed of migrating neural crest and cranial mesoderm cells, which underlies the neural epithelium. The mesenchyme expands and migrates coincidently with neural fold elevation and closure. When migration of cells within that mesenchyme or expansion of the extracellular matrix is disrupted NTC is prevented, suggesting these processes help promote tissue folding^23–25^. Second is the surface ectoderm which is adjacent to the neural epithelium during initial elevation steps. Mutations specific to the surface ectoderm also result in defects in NTC^26–28^. At the very least, this epithelial layer must expand or relax towards the midline to allow the neural folds to move. But we can also envision the surface ectoderm actively pushing in from either side of the neural epithelium, aiding in neural fold elevation and midline bending. You can observe a similar effect by folding a crease into a paper and unfolding it, then placing your fingers at either end of the paper and pushing your fingers towards the center of the paper. Likely, a combination of tissue intrinsic and extrinsic forces working in concert is required for complete NTC.

### Sex differences in early mouse embryo development

In the course of the work presented here, we discovered that female and male mouse embryos potentially exhibit differences in regional tension relative to each other. While females clearly have higher tension laterally than at the midline, males have comparable tension in these two regions. This can be partially explained by deconvolving IRVs on the lateral folds by orientation. Both sexes have high ML IRV, and slightly lower RC IRV, although males fail to reach significance potentially due to their smaller sample size. Male embryos were under-represented in our dataset (70% female, 30% male, Chi Square p = 0.0000830). Male embryos develop faster than females chronologically^29,30^, such that more of them had exceeded the 10 somite stage, beyond which the neural folds are too elevated to ablate with our mounting procedure.

Past work has demonstrated that females have a higher prevalence of cranial NT defects than males^31^, suggesting that sex differences manifest at the latest during NTC. However, at this developmental age, the sex organs have not yet begun to develop and there are therefore no hormonal inputs that could explain these differences^32,33^. Instead, the leading theory points to X-inactivation, which occurs at a much earlier stage of development, as the key genetic difference that may drive a sex bias in NT defects^31^. If there are sex differences in the tissue mechanics of the folding NT, it could help explain why females are more vulnerable to NT defects. For instance, males and females appear to have comparable IRVs in most regions tested, except in the rostral-caudal direction of the lateral neural folds. This is the same axis in which the cranial NT ventrally flexes, a shape that is antagonistic to folding. Higher tension in females in this orientation may indicate a greater antagonism that females need to overcome to compete NTC. However, a full explanation for the sex bias in NT defects, would require understanding how a genetic event like X-inactivation is physically manifested at the cellular, and tissue level.

### Future directions of inquiry

The experiments that we have presented here provide broad insights into the mechanical properties of the cranial mouse NT. However, tension is a complex variable that contains the sum of many factors both internal and external to the neural tissue. One obvious internal candidate is apical constriction on the lateral folds^17^. However, previous studies did not indicate differences between sexes were tested. As our data suggests regional differences in tension anisotropy and regionality between males and females, it would be interesting to determine if this is in part due to differences in apical constriction between sexes. Similarly, as actomyosin often mediates apical constriction, it would be of great interest to determine if there are regional differences in the actomyosin patterning and if this too differs between sexes. We will continue to work to address these and other questions.

## METHODS AND MATERIALS

### Animals

All studies were performed under the United Kingdom Animals (Scientific Procedures) Act 1986 and the Medical Research Council’s Responsibility in the Use of Animals for Medical Research (1993). We obtained outbred time-mated CD-1 mice from the Charles River (CRADL, South Mimms, UK).

### Live Embryo Culture

We set up timed matings of CD-1 mice for a few hours in the morning and the day a plug was identified was designated Embryonic Day (E) 0. We euthanizes pregnant dams by cervical dislocation at E8 and harvested embryos for whole embryo culture as previously described^34^. We dissected embryos in DMEM + 10% fetal bovine serum (FBS) prewarmed to 37°C, leaving the ectoplacental cone, yolk-sac, and amnion intact. We then transferred embryos to vials containing neat, 0.45 μm-filtered rat serum warmed to 37°C and gassed with 5% O_2_, 5% CO_2_ and 90% N_2_. We placed sealed vials into a roller culture incubator for at least 2 hours to allow embryos to acclimate to culture conditions before laser ablation.

### Live Embryo Laser Ablations

For all ablation procedures, we pre-warmed and maintained all medias, dishes, and incubators at 37°C. We dissected, positioned and ablated embryos individually and sequentially using a modified version of a previously developed method^18^. We monitored embryos for a steady heartbeat throughout our procedures as an indication of health. We started by transferring embryos to a 55 mm petri dish containing a 4% agarose-DMEM base submerged in DMEM + 10% FBS. We breached, but left attached, the yolk sac and amnion to reveal the neural tube and transferred embryos to a 1:100 CellMask Deep Red (Invitrogen C10046) in DMEM + HEPES for 3 min to stain membranes. We transferred embryos back to the petri dish and used microsurgical needles from 11–0 Mersilene (TG140–6; Ethicon) and 10–0 Prolene (BV75–3; Ethicon) to position the cranial neural folds upwards and restrict movement. We kept embryos on a Tokai Hit Thermo Plate III (CellSeed) set to 37°C during mounting.

We performed ablations on a Zeiss Examiner LSM880 confocal microscope using a 20×/NA1.0 Plan Apochromat dipping objective with adjustable refractive index collar set to 1.33 and a SpectraPhysics Mai Tai eHP DeepSee multiphoton laser with a nominal power of 1,828 mW (710 nm wavelength, 100% laser power, 4.1 μs pixel dwell time, 0.21 μs pixel size, one iteration). We ablated a 36 μm line (6x the average diameter of cells in the neural folds) on the apical surface of the lateral neural folds or midline along the rostral-caudal or medial-lateral axis the embryo. Ablations instantly vaporized a thin line of tissue^35^. We imaged every 0.416 sec for a total of 20 frames per ablation, with the ablation occurring between frames 2 and 3. We ablated each embryo up to 5 times within a 15 min window after initial breaching of the yolk and amnion, with each ablation occurring in a non-overlapping field of view.

### Embryo Sex Determination and Staging

After ablations, we washed embryos in 1X PBS, dissected and collected the yolk and amnion for DNA extraction. We fixed embryos in 4% paraformaldehyde, pH 7.4, overnight at 4°C. We digested yolk and amnions in 1 mM Proteinase K in Direct PCR lysis buffer (Viagen 301-C) for 1 hr at 55°C to extract DNA. We inactivated samples for 15 minutes at 95°C before using 1 μl of extract for PCR. We determined sex using forward (5′-CTGAAGCTTTTGGCTTTGAG-3′) and reverse (5′-CCACTGCCAAATTCTTTGG-3′), which target *Jarid1c* and *Jarid1d* genes on the X- and Y-chromosome respectively^36^. The primers anneal at 54°C and produce a 331 bp and 302 bp for the X- and Y-chromosomes respectively when separated on a 2% agarose gel.

To score somites, we washed fixed embryos for 10 min in 1X PBS + 0.1% Triton at room temperature. We then incubated them in Phalloidin Alex Fluor 568 (Invitrogen A12380) for 1 hr at room temperature to stain for F-actin. We then washed in 1X PBS + 0.1% Triton for 10 min at room temperature and stored samples in 1X PBS + 1 % NaN_3_. We transferred embryos to a petri dish with a 4% agarose in PBS base submerged in 1X PBS and counted the F-Actin enriched somites on a Leica MZFLIII stereomicroscope.

### Initial Recoil Velocity Analysis

For each ablation, we extracted frames 2 (pre-ablation) and 3 (post ablation) in FIJI. We corrected for noise using the PureDenoise plugin (settings: Automatic – Global, Cycle-spins = 4, Multiframe = 1). We then brought the images into register using the StackReg plugin^37^, set to Rigid Body. To measure initial recoil velocity, we selected points that were 1) clearly visible in both frames, typically cell vertices, 2) abutted opposite edges of the ablation, and 3) were near the center of the ablation path. Orthogonal control points were selected to be adjacent but parallel to the ablation, and parallel control points were outside the ablation path (Figure 1B). We measured a straight-line distance between pairs of points perpendicular to the ablation path. We repeated this measurement 3 times before and 3 times after ablation. We then subtracted the post and pre ablation distances to get 3 values for change in distance, which we averaged into one final value. We divided this average change in distance by the time between frames to calculate initial recoil velocities. We performed all measurements blinded to information regarding the region or orientation of the ablation, embryo sex or age, or the ordering in which the ablations were performed.

We performed a total of 270 ablations across 83 embryos. We removed recoils for any of the following reasons: The ablation was performed in a region outside of the midbrain midline or lateral folds; We were unable to determine the sex or somite stage of the embryo; Bubbles formed in the ablation path; The background embryo had severe movement artifact that prevented accurate measurements. We defined an embryo to have severe movement if the absolute value of the orthogonal or parallel control recoil was greater than the measured initial recoil velocity. A total of 155 ablations across 71 embryos were ultimately used for analysis in this study.

### Statistical Analysis and Figure Assembly

We conducted statistical analyses and graph generation in Python using the Seaborn, Scipy, and Statannotation libraries. We performed an F-test to determine homogeneity of variance between the two groups compared. In instances where the resulting F-test p-value was > 0.05, we performed a t-test assuming equal variance. Otherwise, we conducted t-test assuming unequal variance. We classified values as follows: p-values > 0.05 are nonsignificant (n.s); < 0.05 are shown as “*” on plots; <0.01 were shown as “**” on plots. Neural tube cartoons were sketched in Adobe Illustrator and colored in Adobe Photoshop. All other graphical components were created in Adobe Illustrator. Complete figure panels were assembled in Adobe InDesign.

## Supplementary Material

1
