# Supplementary material for: A lateral tension model for mouse cranial neural tube closure
Source: bioRxiv. 2025 May 15:2025.05.15.654327. Preprint. [Version 1] doi: 10.1101/2025.05.15.654327 (PMC12132523; doi:10.1101/2025.05.15.654327)
Supplement: 1 [file NIHPP2025.05.15.654327V1-supplement-1.pdf]

bioRxiv preprint doi: https://doi.org/10.1101/2025.05.15.654327; this version posted May 15, 2025. The copyright holder for this preprint (which was not certified by peer review) is the author/funder, who has granted bioRxiv a license to display the preprint in perpetuity. It is made available under aCC-BY 4.0 International license.

SUPPLEMENTAL MATERIAL

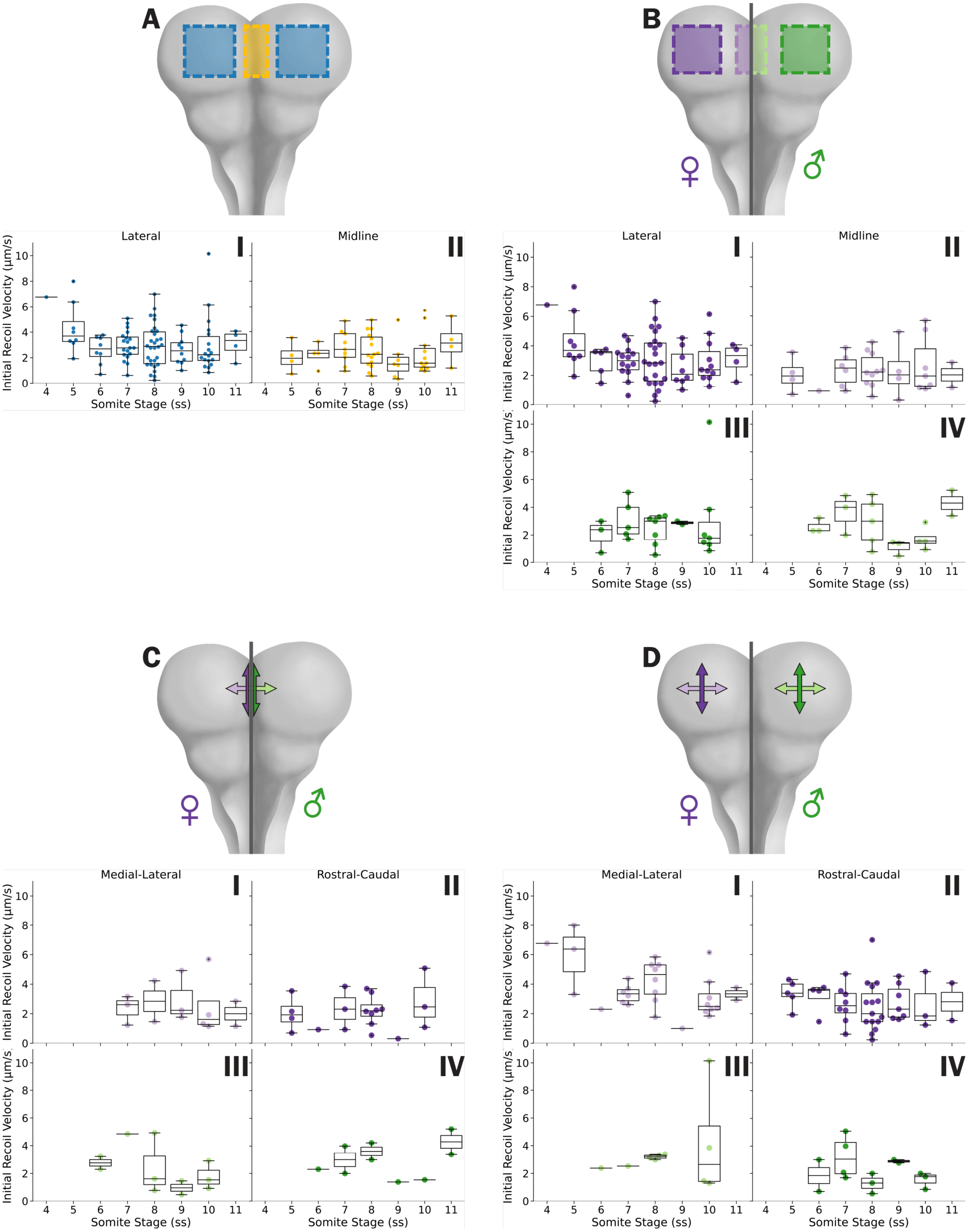

bioRxiv preprint doi: https://doi.org/10.1101/2025.05.15.654327; this version posted May 15, 2025. The copyright holder for this preprint (which was not certified by peer review) is the author/funder, who has granted bioRxiv a license to display the preprint in perpetuity. It is made available under aCC-BY 4.0 International license.

Supplemental Figure 1: Initial recoil velocities show no difference over developmental time. **A-D)** A cartoon schematic of location where initial recoil velocities were measured for their respective subplots (**I-IV**). **A** and **B)** Shaded areas indicate regions where initial recoil where measured. **C** and **D)** Double headed arrows indicate the direction inferred tension was measured in the **C)** midline and **D)** lateral neural folds. In **B-D)** female measurements are in purple hues and males are in green hues. In **I-IV)** box plots show the median and interquartile range.
